# Is it the road or the fence? Influence of linear anthropogenic features on the movement and distribution of a partially migratory ungulate

**DOI:** 10.1186/s40462-022-00336-3

**Published:** 2022-08-29

**Authors:** Paul F. Jones, Andrew F. Jakes, Scott E. Vegter, Mike S. Verhage

**Affiliations:** 1 Alberta Conservation Association, #400 817-4th Ave South, Lethbridge, AB T1J 0P3 Canada; 2 Smithsonian’s National Zoo and Conservation Biology Institute, 1500 Remount Road, Front Royal, VA 22630 USA

**Keywords:** Alberta, Anthropogenic linear feature, Barrier effect, Crossing effect, Fences, Movement tactic, Pronghorn, Proximity effect, Relative selection strength, Roads

## Abstract

**Background:**

Anthropogenic linear features change the behavior and selection patterns of species, which must adapt to these ever-increasing features on the landscape. Roads are a well-studied linear feature that alter the survival, movement, and distribution of animals. Less understood are the effects of fences on wildlife, though they tend to be more ubiquitous across the landscape than roads. Even less understood are potential indirect effects when fences are found in tandem with roads along transportation corridors.

**Methods:**

We assessed how the spatial configuration of fences and roads effect the movement (crossing effect) and distribution (proximity effect) of a partially migratory pronghorn population (*Antilocapra americana*) on the grasslands of southern Alberta, Canada. We used data from 55 collared pronghorn within a step-selection function framework to assess the influence of 4 linear features: (1) pasture fences, (2) roads not fenced, (3) roads fenced on one side, and (4) roads fenced on both sides on the selection pattern of migratory and resident animals. We examined whether steps along a movement pathway (i.e., crossing effect) were influenced by the type of linear feature animals attempted to cross and, whether these features affected the distribution of pronghorn (i.e., proximity effect) across the landscape.

**Results:**

The top model for crossing effect for both movement tactics contained all 4 linear features and land cover. Regression coefficients were negative for all linear features, indicating that individuals were less likely to chose steps that crossed linear features. For the proximity effect, migrant animals avoided all linear features except roads fenced on both sides, where they selected areas closer to this feature. Resident animals, on the other hand, were found closer to pasture fences but further from roads without fences.

**Conclusions:**

Our results indicate that both fences and roads are indirectly affecting pronghorn resource use spatially and behaviorally, whether each linear feature is found separately or in tandem. Modifying existing fences and roads to account for responses to these distinct linear features could facilitate more successful crossing opportunities and/or shifts in distribution. Allowing pronghorn to freely move across the landscape will maintain functional connectivity to ensure population persistence of this endemic ungulate.

**Supplementary Information:**

The online version contains supplementary material available at 10.1186/s40462-022-00336-3.

## Introduction

Anthropogenic development and features are quickly changing the face of terrestrial ecosystems. It is estimated that human activities have altered 50–70% of the world’s surface, resulting in changes to biodiversity and ecosystem function [1]. Anthropogenic features and their ever-increasing densities on the landscape [2, 3] result in direct and indirect habitat loss [4, 5], behavioral changes [6, 7], and increased mortality [8, 9] for wildlife. Consequently, anthropogenic features have been implicated in the loss of daily and seasonal movements, especially for migratory ungulates [1].

While we can measure habitat loss and quantify mortality from anthropogenic features, it is more difficult to evaluate the indirect influence these features may have, e.g., in terms of a behavioral response (e.g., avoidance). This is especially true for linear features, such as roads, railroads, seismic lines, power lines, and fences, that may impact wildlife behaviour, and act as barriers to movement and can result in decreased populations [10–12]. The negative effect of movement barriers manifest themselves in two ways: (1) barriers can increase the energetic costs on a daily and seasonal basis, thus potentially increasing mortality risk and may result in a loss in fitness, (2) barriers can alter animal behavior and ultimately reduce crossing probability, resulting in the potential loss of available habitat at an individual level and a reduction over time in the number of individuals migrating between habitat patches [12]. To fully capture and quantify the effects of linear features, research must assess impacts not only from a habitat loss and mortality perspective, but also measure behavioral responses (crossing effect and proximity effect; [13]). As defined by Beyer et al. [13], crossing effect is the assessment of the permeability of the barrier while proximity effect is the probability of space use as a function of the distance to the barrier. Accounting for wildlife behavioral responses allows managers to recognize the potential avoidance of quality habitat caused by linear features [14, 15].

Two common anthropogenic linear features on the landscape are roads and fences. Roads are the most studied linear feature and their detrimental impacts to wildlife and ecosystems are well documented [16]. These studies have led to the development of ‘road ecology’; a discipline specifically designed to study and understand the effects that roads have on wildlife and ecological processes [16–18]. Road type varies based on surface and traffic volume and often parallel fencing is erected, especially along paved, high-speed, high-traffic volume roads, to reduce the potential of wildlife-vehicle collisions [19, 20]. While roadside fencing is effective at reducing wildlife-vehicle collisions, it often exacerbates the barrier effect within a transportation corridor [21]. In addition to roadside fencing, fences are erected to mark boundaries (i.e., border or property), and as a tool to control the distribution of domestic livestock and wildlife throughout the world [22, 23]. Fences take on many forms including multi-strand barbed wire fence to page wire, and are more widespread across the landscape than roads [22, 23]. The effects of fences on wildlife are poorly understood in comparison to roads, though ‘fence ecology’ as a research field is gaining momentum [22, 23]. Even less understood are the potential increased behavioral impacts on wildlife when fences are found within close proximity to roads.

Linear features such as roads and fences create different challenges for wildlife as each feature varies spatially and in permeability [14]. Both roads and fences have proven to be barriers affecting wildlife movement and space use [24–26]. How a species responds to linear features will be governed by the feature’s physical characteristics, the species’ behavioral adaptability to the disturbance, its mobility, and resource requirements. Understanding the mechanisms of how linear features impact the movement (i.e., facilitates or impedes) and distribution (i.e., use of space) of a species is required if we are to effectively manage populations [14]. For example, if wildlife continually approach linear features but are unable to cross, it may appear that they are selecting areas in proximity to the linear feature. Therefore, understanding how wildlife selection patterns are influenced by linear features at multiple scales [27] is required. At a fine scale, a linear feature may affect a species’ ability to navigate successfully (i.e., crossing effect), while at a broader scale the feature may affect how animals distribute themselves on the landscape (i.e., proximity effect) in relationship to the feature [14]. In addition, not only accounting for the spatial configuration between fences and roads, but also the varying densities of these configurations across the landscape will provide insight into how permeable the landscape is for movement by wildlife.

**Fig. 1:**
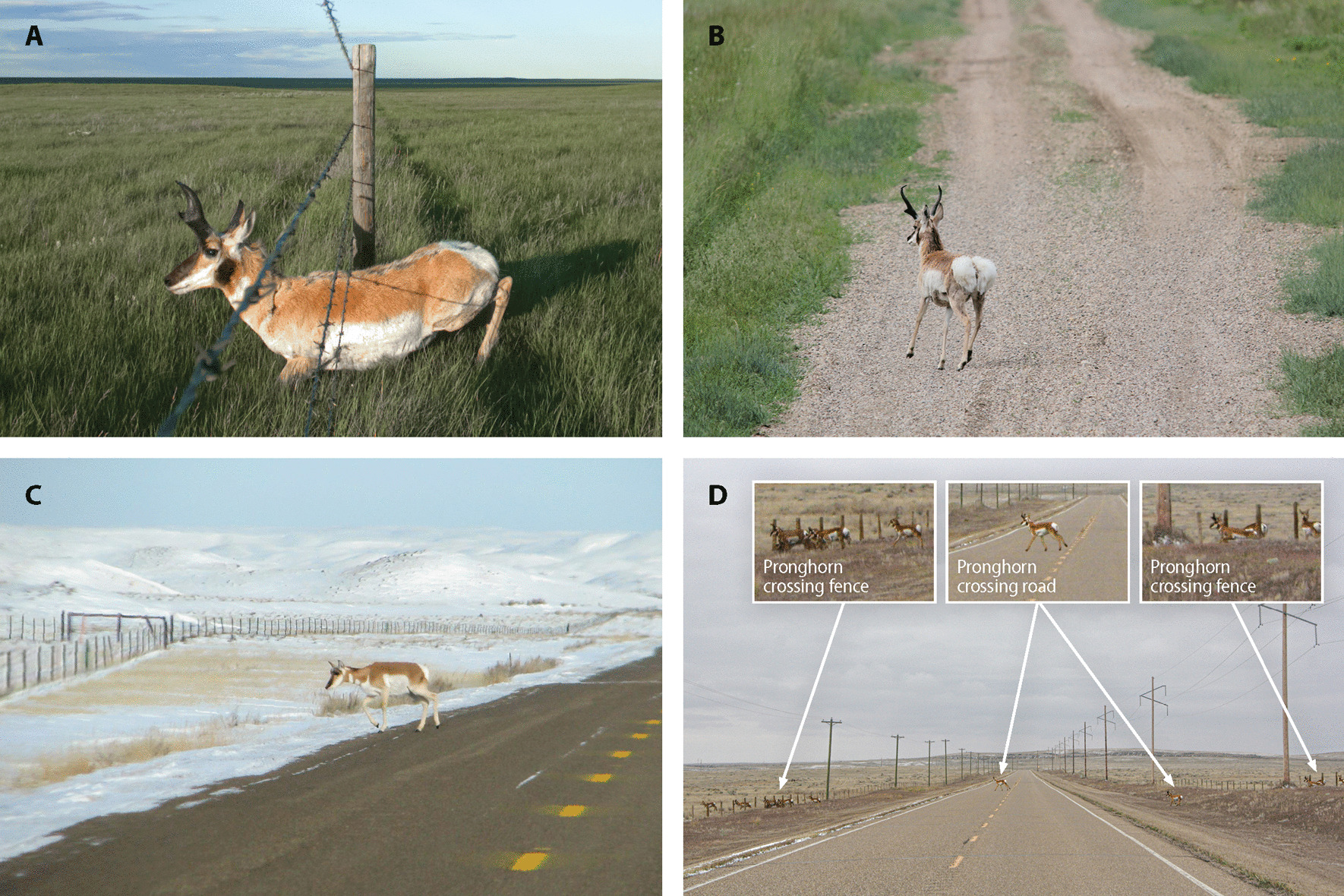
Example of the 2 different crossing behaviors exhibited by pronghorn when crossing a fence (**A**) and a road (**B**). When crossing under a fence, a pronghorn must crawl on its elbows to get under the bottom wire of the fence (**A**). Note the hair loss and scaring on the neck and back of the animal depicted in (**A**) as a result of crawling under fences. When crossing a road, a pronghorn can walk or run across depending on the presence of a vehicle (**B**). Note the flared white rump patch on the animal depicted in (**B**), which is an anti-predatory alarm sign used by pronghorn. Panel **C** depicts a pronghorn crossing a road that is fenced on one side. Panel **D** depicts a group of pronghorn crossing a road with two fences where both the fence and road crossing behaviors are employed. Also note the spatial distance covered to successfully cross each linear feature. (Photos: **A**—Alberta Conservation Association; **B**—P. F. Jones, Alberta Conservation Association, **C**—A. F. Jakes, Smithsonian Conservation Biology Institute, **D**—A. MacDonald, Alberta Conservation Association)

Pronghorn (*Antilocapra americana*) are an endemic ungulate to the plains of North America and exhibit migratory behavior. Most pronghorn populations are partially migratory, with some individuals (migrants) moving between distinct summer–winter ranges while others (residents) remain in the same general area year-round [28–30]. The direct and indirect impact of roads on pronghorn are well-documented [8, 31–33]. Fences also have direct [8] and indirect [5, 34, 35] impacts on pronghorn. Our understanding of how pronghorn respond to the spatial configuration of roads and fences (i.e., roads that are fenced on both sides, fenced on one side, or not fenced, and pasture fences not associated with roads) is lacking and needs attention to effectively mitigate barriers to movement. Due to variable fence-road spatial configurations (Fig. 1A–D), pronghorn likely alter their response when trying to navigate isolated features (e.g., pasture fence) or when found in tandem (e.g., fenced road). Pronghorn are reluctant to jump fences and typically cross by crawling under the bottom wire in a single file fashion [36]. Depending on whether vehicles are present, pronghorn will walk or run (reaching speeds of up to 100 km/h) across roads. Pronghorn will employ one or both crossing behaviors to account for the spatial configuration of the linear feature being crossed (Fig. 1A–D). In addition, the length of time and distance covered by an individual when crossing will vary depending on the configuration of the linear feature. Working in the same area of Alberta, Gavin and Komers [31] showed pronghorn to perceive roads equivalent to a predation risk and shift from foraging to a vigilant state when in proximity. However, no data was collected on whether the roads assessed were fenced or not, and how this influenced their results. We expand on the work of Gavin and Komers [31] by accounting for potential differences in behavioral responses associated with the spatial configurations of the two linear features.

Our objective was to assess the relative impacts of two common linear features on the crossing and proximity effect on pronghorn [14]. Our intent was not to develop an all-encompassing resource selection function model. Rather, we assessed how fences, roads with fences, and roads without fences influences the crossing effect (i.e., behaviour) and proximity effect (i.e., distribution) of migrant and resident pronghorn. In addition, we aim to demonstrate how the spatial configuration between different linear features can provide greater insight into behavioral responses to anthropogenic disturbances. To account for the spatial configuration between roads and fences, we treated each possible combination of the two features as separate covariates in our models. The 4 covariates were: (1) fences not associated with roads, (2) roads with no fences, (3) roads fenced on one side, and (4) roads fenced on both sides (Fig. 1). For the crossing effect, we assessed the permeability of each linear feature, measured as the number of crossings, to determine its influence on the ability of pronghorn to successfully cross. We predict that each covariate would be retained in the top model and that each would have varying degrees of permeability to movement. Therefore, we predicted that if each linear feature were semi-permeable, the selection strength by pronghorn would decrease as the number of crossings of each feature increased. For the proximity effect, we examined the distribution of pronghorn on the landscape, measured as ‘distance to’ each linear feature. For the proximity effect, we predicted that all 4 covariates would be retained in the top model and that pronghorn would show avoidance towards each feature. The predictions for the crossing and proximity effects were based on the fact that both fences and roads have been shown to negatively affect pronghorn movement and distribution [5, 31, 32, 35–37]. Lastly, as migrating pronghorn move between distinct seasonal ranges, we predicted that migratory individuals would have a greater exposure to linear features and therefore show higher crossing rates and be found more often within proximity of linear features compared to resident individuals [11, 32, 38, 39].

## Methods and materials

### Study site

Our study occurred within the grassland region of Alberta, Canada (50.5167°N, – 111.2445°W) within a large area where spatial data of fences were available (Additional file 1: Fig. S1). The study area is 59534 km^2^ in size and is part of a larger region termed the Northern Sagebrush Steppe (NSS) that includes areas in Alberta and Saskatchewan, Canada, and Montana, USA (Additional file 1: Fig. S1). The landscape is characterized by flat, open plains and rolling hills as a result of glacial recession and deposits, with prevalent badlands and deep coulees throughout the region [40]. Human populations are sparsely distributed with few urban population centers (Additional file 1: Fig. S1). Cattle grazing is the dominant land use with oil and natural gas wells occurring at high densities. The region is considered semi-arid and received an annual mean of 39.2 cm of precipitation, with approximately 70% as rainfall [41].

### Pronghorn data

We captured adult female pronghorn in a mixture of native and cultivated areas using a net fired from a helicopter [42] between 2003 and 2007. Each captured female was fitted with a Global Positioning System (GPS) collar (Lotek GPS 3300; Lotek Wireless, Newmarket, Ontario, Canada) and released as quickly as possible to reduce risk of capture myopathy [36]. Each GPS collar recorded a relocation every 4 h and was equipped with a mortality sensor set at 4 h. Collars were programmed to drop-off following 50–52 weeks, were retrieved in the field, and data downloaded from the collar before it was redeployed on a new individual. The data collected covers 2003–12–12 to 2004–11–23, 2005–03–03 to 2006–02–24, and 2006–03–03 to 2007–02–28. Capture protocols were approved by Alberta Fish and Wildlife (Alberta Sustainable Resource Development, Fish and Wildlife Research Permits 11861, 16707, and 20394). Additional details on capture protocol are provided in Jones et al. [43].

We used the results of Jakes et al. [30] to assign movement tactic (i.e., migratory or resident) to individual pronghorn. Initially, movement tactic was assigned to each pronghorn based on non-linear models of net squared displacement [44] that classified migration periods and categorized individuals into migratory, mixed-migratory, or resident. For our study, we reduced the categories to 2 by combining individuals in the mixed-migratory and migratory classes into 1. Migratory animals were defined as individuals that moved between 2 distinct seasonal ranges in a biological year while residents remained year-round on a single range.

### Spatial data

We used spatial data from 2 sources to construct our linear features. We used a southeast Alberta fence layer developed from high resolution aerial imagery [45], and the Alberta portion of the NSS road network that was derived as part of a larger project [46]. The fence layer was constructed by digitally mapping characteristics associated with fence lines (i.e., domestic livestock trails) on imagery collected between 1999 and 2001 [45]. The fence layer provided spatial data on fence location but no information on fence design, as this is difficult to assess from aerial imagery [22]. However, most fences in Alberta are 3–5 strand barbed wire fences that are used to control the distribution of livestock. Though some changes to existing fence lines may have occurred between when the imagery was taken and when our GPS collar data was collected, they likely are minor in scale and therefore would not impact our results. The NSS road layer was constructed in 2011 and for the AB portion, used road data provided by Alberta Sustainable Resource and Development through a partnership with AltaLIS Ltd., an agent for Spatial Data Warehouse Ltd [47]. Roads were originally categorized into 3 classes based on surface type, number of lanes, and traffic volume. The three classes were paved divided (major highway with high-speed, high traffic volume), paved undivided (secondary highway with medium to large traffic volume) and gravel, dirt, or truck trail (county roads with low traffic volume). We combined all the road categories into one under the assumption that a barrier effect of roads would be constant for all road types and traffic volumes [48]. We buffered major and secondary highways by 40 m and county roads by 20 m to determine if the road was fenced or not. The 40 m buffer represents the standard right-of-way area for paved roads in Alberta, while the 20 m buffer represents the standard right-of-way area for unpaved roads (Additional file 1: Supplementary Information 1). We clipped the road layer to the extent of the fence data to explore and build layers which accounted for the different configurations between roads and fences (see Additional file 1: Supplementary Information 1 for details on layer creation steps). We used ArcGIS Desktop 10.6.1 [49] for layer construction. The final 4 linear features that captured the different configurations and used to model crossing and proximity effects were: (1) pasture fence (hereafter FENCE), (2) road no fence (hereafter RNF), (3) road fenced on one side (hereafter R1F), and (4) road fenced on both sides (hereafter R2F). The average width of a fence, RNF, R1F, and R2F were 1 m, 16.5 m, 25.8 m, and 41.7 m respectively.

### Choice set and covariates

We applied a used-available resource selection function design [50] that compared used (i.e., pronghorn GPS relocation) and available point locations to estimate relative probability of use in a multi-scale analysis. As we were specifically interested in the effects of fences and roads on pronghorn, we only used these covariates in our models along with a base model that consisted of land cover. We used land cover as our base model to account for the known selection by pronghorn for native prairie [36] and to determine if the inclusion of fences and roads improved model fit. Land cover covariates were derived from the land cover for agricultural regions of Canada [51] dataset. We reduced the number of categories of the land cover covariate to 4 by combining categories. The 4 categories were ‘NATIVE’ (combined categories native prairie, shrub, and wetland), ‘ANNUAL CROP’, ‘PERENNIAL’, and ‘OTHER’ (combined categories deciduous tree cover, coniferous tree cover, mixed tree cover, exposed land, developed land, and water). We only used female pronghorn that had a minimum of 500-relocations. We retained animals in our dataset, either resident or migratory, that ventured outside of our study area as long as they had the minimum 500-relocation within the study area. We set the 500-relocation minimum, similar to that set by [25], to ensure model estimates were stable. We completed our analysis separately for migratory and resident individuals.

**Fig. 2:**
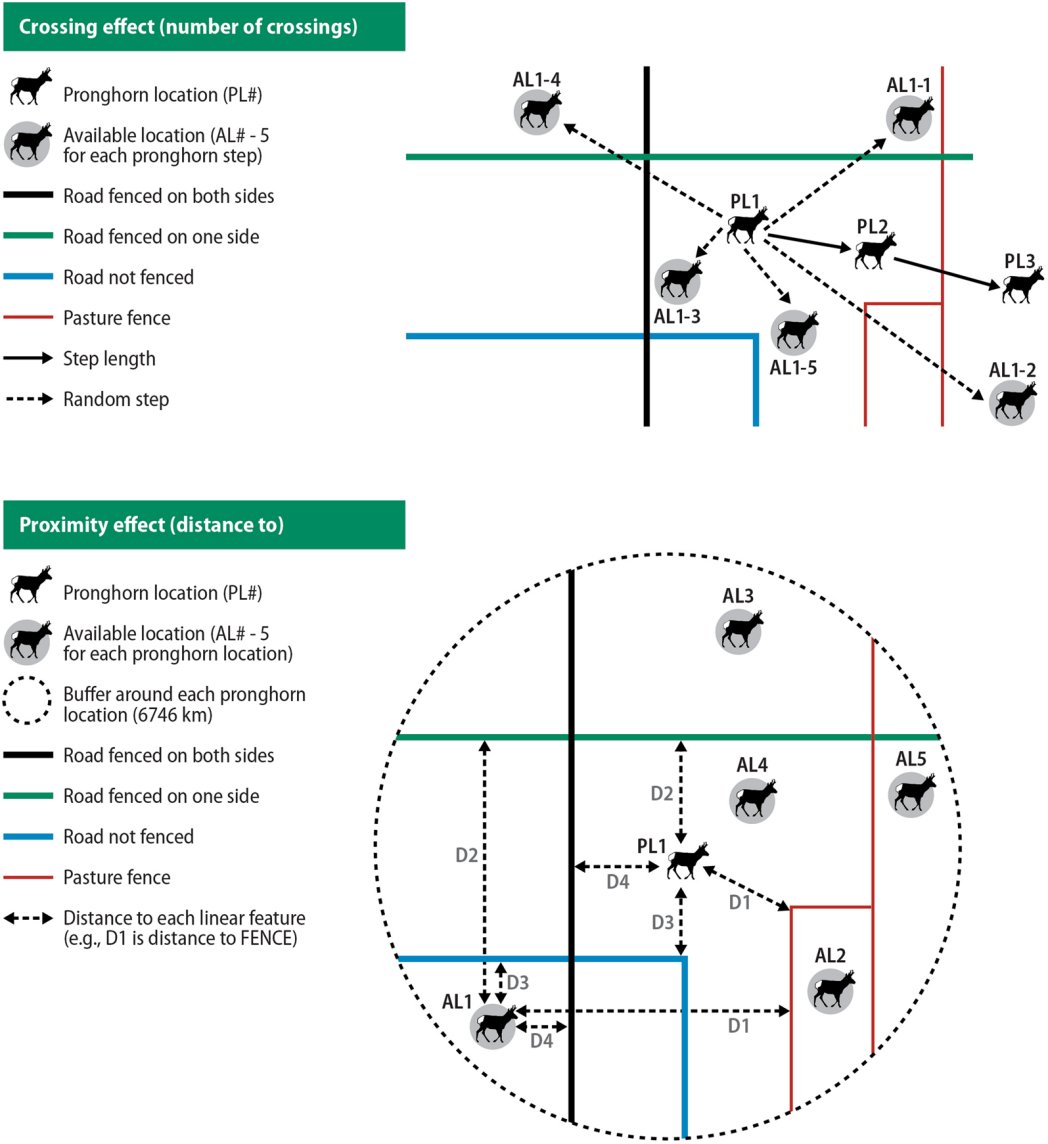
Conceptual diagram of the sampling design for the crossing and proximity effects for migrant and resident pronghorn in the Alberta, Canada, 2003–2007. For the crossing effect, we calculated step lengths and turning angles between consecutive used Global Positioning System points and used those distributions to randomly select five step lengths and five turning angles for each used point. We then counted the number of crossings of each of our 4 linear features between successive used locations and between the used location and its paired five available points. For the proximity effect, we buffered each used point by 6746 km (sensu [5]) and then randomly placed 5 available points within this buffer. We then calculated the distance to the nearest feature for each linear feature. The 4 linear features were pasture fence (FENCE), a road with no associated fences (RNF), a road fenced on one side (R1F), and a road fenced on both sides (R2F)

We completed our analysis at 2 spatial scales based on how the available data were generated: crossing effect at the fine scale and proximity effect at a broader scale. We generated 5 available points for each relocation at both scales (Fig. 2). For the crossing effect, we used a step-selection function framework [52] and calculated the distribution of step lengths and turning angles between consecutive used points and applied those distributions to generate 5 random step lengths and 5 random turning angles for each used point. The distributions of step length and turning angles were specific to each individual to avoid issues of circularity [52] and allow for the evaluation of selection between movement tactics. For the proximity effect, we buffered each used point with a circular 6746 m buffer, sensu Jones et al. [5], and randomly generated 5 available points within the buffer (Fig. 2). The buffer size ascertained by Jones et al. [5] was calculated as the 90^th^ percentile from the step length distribution, identified by randomly selecting 1 used location every 4 days and connecting locations. The individuals used for our analysis were part of the data set used to calculate the identified 6746 m buffer size and therefore appropriate for this study. Hereafter, we use ‘choice set’ to indicate a used point and its corresponding available points [53].

For the crossing effect, we developed crossing covariates by drawing a line (i.e., step lines) from each used and available point for a given choice set back to the previous used point. We used the number of times each linear features was crossed by an individual relative to the number of times each individual could have crossed during each step in our analysis. We removed relocations from the analysis that did not have a relocation either 4 h prior or after the relocation to ensure all step lengths occurred over a 4-h time period. We examined the distribution of data and determined that pronghorn relocations were distributed closer to 0 crossings, while the available points had more crossings. This difference in distribution between pronghorn relocations and available points caused issues with convergence in the logistic regression. Therefore, we determined the approximate 98th percentile of crossings of each of the covariates and used these values to cap the number of crossings (see Additional file 1: Table S1). For the proximity effect, we assessed the distribution of pronghorn (i.e., used relocation) compared to where pronghorn could have distributed themselves (i.e., available points) across the landscape using a distance metric. We first calculated the distance from each pronghorn relocation and available point to the nearest feature for all 4 linear features. We capped distances to 1000 m, a distance where pronghorn across this region have previously shown selection at [31, 47].

### Data analysis

We fit all models across animals and movement tactic (migratory and resident). For both analysis we applied a conditional logistic regression analysis using the Cox-proportional hazards approximation in the *Survival* R package [54]. Prior to modeling, we performed a Pearson’s pairwise correlation analysis to identify any potential collinearity issues; none of the covariates considered for the crossing or proximity effects exhibited strong correlation (|r|> 0.7). For both the crossing and proximity effects, we examined the regression (β) coefficients of the covariates and their standard errors during the modeling process for large changes in coefficient estimates coinciding with the addition of a particular covariate which can indicate multi-collinearity [55]. We evaluated the support among competing models using AIC to assess model fit [56] and compared all possible combinations of the 4 linear features, land cover, plus the null model. We used ΔAIC < 2.0 as the initial cut‐off to compare competing top models and then examined the covariates and model weights in each competing model to determine if including additional covariates improved model performance [57]. For both the crossing and proximity effects, after top models were selected, we standardized continuous covariates using the Gelman [58] approach and refitted the models to aid in the direct comparison of covariates.

For both the crossing and proximity effects, to interpret the effects of the covariates in the top models, we used the approach of Fieberg et al. [59] to calculate the relative use of a location compared to a second location across the range of each covariate. We calculated the log-relative intensity which is referred to as the log-Relative Selection Strength (log-RSS) by Avgar et al. [60], using the *amt* package in R [59]. Unless otherwise stated, we set the other covariates to their mean and land cover to NATIVE when completing the comparisons. For the covariates in the top model, for the crossing effect, we were particularity interested in the relative selection strength (RSS as the exponential of the log-RSS; [59]) when pronghorn decided not to cross (crossing rate = 0) compared to when they decided to cross (crossing rate = 1). For each covariate, we set the number of crossings to 0 and 1 in location 1, and the number of crossings to 0 in the second location, while holding the other linear features for both locations constant at 0 [59]. We also calculated the RSS by comparing the number of crossings across the range of values for each covariate (location 1) to the mean number of crossings of that covariate (location 2). For the covariates in the top model for the proximity effect, we calculated the RSS by comparing the distance across the range of values for each covariate, to the mean distance of that covariate [59]. All analysis was completed in R version 4.0.3 [61] and R Studio version 1.3.1093 [62]. For all data summaries and analysis, unless otherwise specified, we used the respective capped values.

## Results

We respectively captured 24, 25, and 25 female pronghorn in December 2003, March 2005, and March 2006 within our study area. Of these 74 captured animals, we used data from 55 females in our analysis and of these, 24 were identified as migratory and 31 were resident. For the crossing effect analysis of migratory animals, the mean number of relocations was 1568.04 (SE = 110.02, range = 503–2173), while for residents the mean was 1825.81 (SE = 75.10, range = 576–2169). For the proximity effect, the mean number of relocations for migratory animals was 1577.83 (SE = 110.62, range = 513–2174) and for residents it was 1836.03 (SE = 74.80, range = 585–2172). For the crossing effect the sample sizes represents consecutive relocations and are lower than the sample sizes for the proximity effect which include all relocations within the study area. For the 4 linear features the density in the study area was 0.71 km/km^2^ (42,296 km), 0.50 km/km^2^ (29,484 km), 0.18 km/km^2^ (10,423 km), and 0.11 km/km^2^ (6275 km) of FENCE, RNF, R1F, and R2F respectively.

### Crossing effect

**Fig. 3:**
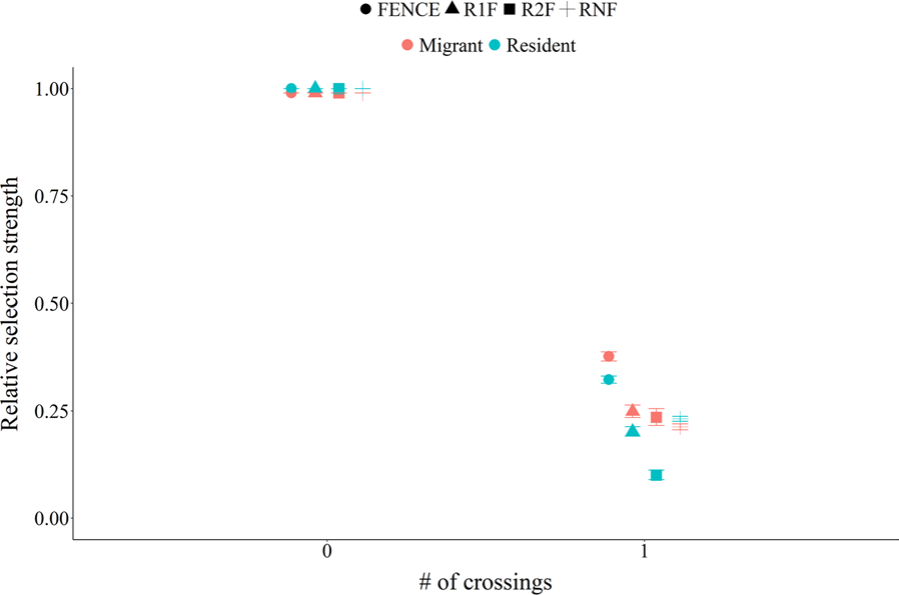
Relative selection strength (exponential of the log-RSS) for the covariates in the top conditional logistic model for the crossing effect for migrant and resident pronghorn in Alberta, Canada, 2003–2007. The RSS compared the number of crossings set at 0 and 1 to the number of crossings set at 0 of that linear feature, while holding the other linear features in the model constant at 0 and setting land cover to NATIVE. The 4 linear features were pasture fence (FENCE), a road with no associated fences (RNF), a road fenced on one side (R1F), and a road fenced on both sides (R2F). Note points were jittered horizontally and vertically to make them easier to visualize

For the crossing effect, both migratory and resident animals had top models containing land cover and all 4 covariates representing the linear features (Additional file 1: Table S2). For both movement tactics, the other models evaluated had ∆AIC values > 2.0 and were not considered competitive. For the crossing effect, the covariates were measured as the number of crossings of each linear feature. All standardized regression (β) coefficients were negative (Table 1) indicating that both movement tactics avoided crossing all linear features. Both movement tactics used NATIVE more and OTHER less than ANNUAL CROP (Table 1). For migrant animals there was a 62%, 79%, 75%, and 77% decrease in the RSS between not crossing and crossing once for FENCE, RNF, R1F, and R2F, respectively (Fig. 3). For resident animals there was a 68%, 77%, 80%, and 90% decrease in the RSS between not crossing and crossing once for FENCE, RNF, R1F, and R2F, respectively (Fig. 3). Additional file 1: Fig. S2 shows the RSS across the range of capped crossing values for both migrant and resident animals for the 4 linear features.

**Table 1:** Crossing effect selection standardized regression coefficients and standard error (SE) for the covariates within the top conditional logistic regression model for migratory and resident pronghorn in Alberta, Canada, 2003–2007

Covariate	Migrant		Resident	
	Regression coefficient	SE	Regression coefficient	SE
FENCE	− 4.61	0.01	− 3.54	0.03
RNF	− 4.04	0.02	− 3.66	0.03
R1F	− 2.25	0.11	− 1.69	0.18
R2F	− 1.66	0.19	− 1.35	0.26
NATIVE	0.27	1.30	0.21	1.23
OTHER	− 0.94	0.39	− 1.09	0.34
PERENNIAL	0.12	1.13	− 0.73	0.48

For the 4 linear features the values represent the number of crossings capped at the approximate 98% percentiles. The 4 linear features were pasture fence (FENCE), a road with no associated fences (RNF), a road fenced on one side (R1F), and a road fenced on both sides (R2F). The land cover types NATIVE, OTHER, and PERENNIAL were in comparison to the reference category ANNUAL CROP

### Proximity effect

**Fig. 4:**
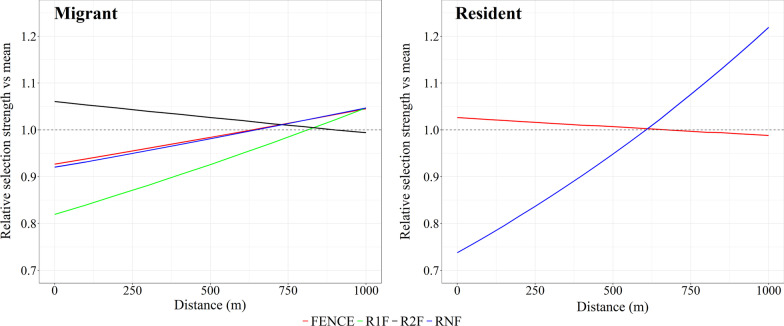
Relative selection strength (exponential of the log-RSS) for the covariates in the top conditional logistic model for the proximity effect for migrant (left graph) and resident pronghorn (right graph) in Alberta, Canada, 2003–2007. The RSS was calculated by comparing the distance (m) across the range of values for each covariate to the mean distance of that covariate, while holding the other covariates in the model constant at their mean and setting land cover to NATIVE. The 4 linear features were pasture fence (FENCE), a road with no associated fences (RNF), a road fenced on one side (R1F), and a road fenced on both sides (R2F). Note each covariate was capped at 1000 m

For the proximity effect, migratory animals had a top model that contained land cover and all 4 covariates that represented the linear features, with the next competing model having a ∆AIC > 2.0 (Additional file 1: Table S2). The top model for migratory animals had 78% of the model weight. For resident animals there were 4 competing models with the competing models having a model weight of 78% (Additional file 1: Table S2). All other models had ∆AIC > 2.0. The resident top model contained the covariates land cover, distance to FENCE, and distance to RNF and had 31% of the model weight (Additional file 1: Table S2). For the proximity effect, the covariates represent distance to each linear feature, with differences in selection between migratory and resident animals detected (Table 2). The regression coefficient for distance to FENCE was positive for migratory animals and negative for resident animals, while all three regression coefficients for the land cover categories, in reference to ANNUAL CROP, were negative for resident animals and only negative for OTHER for migratory animals (Table 2). Similarities between migratory and resident animals occurred for the covariate RNF whose regression coefficient was positive. For migrant animals there was an increase of 13%, 14%, 28%, and decrease of 6% in the RSS between being close (0 m) or far (1000 m) from a FENCE, RNF, R1F, and R2F, respectively (Fig. 4). For resident animals, using just the top model from the 4 competing models, there was a decrease of 4% and a 65% increase in the RSS between being close (0 m) or far (1000 m) from a FENCE and RNF, respectively (Fig. 4).

**Table 2:** Proximity effect selection standardized regression coefficients and standard error (SE) for the covariates within the top conditional logistic regression model for migratory and resident pronghorn in Alberta, Canada, 2003–2007

Covariate	Migrant		Resident	
	Regression coefficient	SE	Regression coefficient	SE
FENCE	0.09	0.02	− 0.03	0.012
RNF	0.09	0.01	0.35	0.011
R1F	0.14	0.02	n/a	n/a
R2F	− 0.03	0.01	n/a	n/a
NATIVE	0.11	0.02	− 0.09	0.015
OTHER	− 0.68	0.06	− 0.91	0.041
PERENNIAL	0.02	0.03	− 0.52	0.021

For the linear features the values were capped at a maximum distance to of 1000 m. The 4 linear features were pasture fence (FENCE), a road with no associated fences (RNF), a road fenced on one side (R1F), and a road fenced on both sides (R2F). The land cover types NATIVE, OTHER, and PERENNIAL were in comparison to the reference category ANNUAL CROP

## Discussion

Studies that have quantified the influence of anthropogenic features on wildlife have mainly examined effects from the direct loss of habitat [4, 15]. Fewer studies have examined the indirect effects of anthropogenic features on wildlife, particularly from a crossing and proximity effect [14, 63]). Here we disentangle the crossing and proximity effects from the spatial configuration of two linear features on a partially migratory species. Our results clearly demonstrate the spatial and behavioral impact that both fences and roads have on the ability of pronghorn to move and distribute themselves freely across the landscape. Both movement tactics showed a dramatic decrease in selection when faced with having to cross any of the 4 linear features (range: 62–90% reduction). Our assessment of the proximity effect exhibited a trend for pronghorn to be found further from linear features. Even in the case of unfenced roads pronghorn from both movement tactics were found to exhibit avoidance of this linear feature. The avoidance behavior exhibited by pronghorn during our study is consistent with previous findings for a suite of ungulates [25, 31, 33, 64]. Two exceptions were that migrant animals were found closer to roads fenced on both sides, and that resident animals were found closer to pasture fences. Although we aimed to identify which linear feature had the greatest impact, our results suggest that all 4 features play a role and influence the crossing and distribution of pronghorn. Therefore, each of these features need to be accounted for in conserving ungulate daily and seasonal movements.

Examining the crossing effect revealed pronghorn crossed pasture fences more often than the other 3 linear features as this feature was more prevalent on the landscape. Crossings were higher for pasture fences than any of the other linear feature (Additional file 1: Table S1) for pronghorn using either movement tactic. While pronghorn cross pasture fences more frequently, one should not ignore their adverse effects on crossing behavior as evident by the strong negative relationship. For example, selection decreased by over 60% if an animal, using either movement tactic, had to cross a pasture fence and we interpret this as impacting a pronghorn’s ability to freely move across the landscape. Pronghorn and mule deer (*Odocoileus hemionus*) in Wyoming also experienced difficulties with crossing fences, with over 40% of fence encounters by both species resulting in altered movement behavior [64]. Further, Xu et al. [64] demonstrated that pronghorn tended to ‘bounce’ away when encountering a fence. Our results provided further evidence of a fence barrier effect for pronghorn [5, 64], which has been observed for other wildlife species [26, 65–67].

Understanding the impacts of anthropogenic features on the persistence of migration in mammals is at the forefront of conservation [1, 38]. Identifying similarities and differences in selection patterns between movement tactics employed by partially migratory species is key. Passoni et al. [68] found no differences in avoidance of roads between migrant, resident, and dispersing roe deer (*Capreolus capreolus*). In contrast, Sawyer et al. [69] found that long-distance migrant mule deer in Wyoming crossed more roads and fences compared to short- and medium- distance migrants. They attributed the different crossing decisions were a result of the large distances covered by migrants [69]. We assessed the selection patterns by migrants and residents and note two very contrasting results between movement tactics. First, migrant animals selected areas closer to roads fenced on both sides, while for resident animals, roads fenced on both sides was not in the top model. Animals classified as migrants traversed the study area and covered greater distances than resident animals. Pronghorn in the NSS make one of the longest known migrations across North America, moving on average 162 km, while one individual moved over 800 km in a year [30]. Therefore, migrant animals were likely impacted more often (i.e., had to cross) with this linear feature. The fact that migrants selected areas adjacent to roads fenced on both sides is an artifact of their movement patterns and reveals that this linear feature is a semi-permeable barrier, with animals ‘piling up’ along roads fenced on both sides before discovering locales to successfully cross and continue on their original movement trajectory. The ‘pilling up’ of animals makes it appear they are selecting areas closer to roads when in fact the road fenced on both sides is acting as a semi-permeable barrier and hindering movement. Roads in Norway were also identified as semi-permeable linear features as they restricted the ability of moose (*Alces alces*) to cross [2]. Elk (*Cervus elaphus*) while able to physically cross a road, tended to show selection against crossing them in the foothills of Alberta, Canada [25]. However, neither study indicated whether the roads being traversed by these ungulates were fenced or not, which likely influenced the underlying mechanisms behind selecting against roads and overall study inferences. This in a common deficiency as many studies identifying roads as problematic for wildlife have not indicated if the roads under question are fenced or not (exceptions are studies designed to assess roadside fences designed to reduce animal-vehicle collisions). Our results clearly indicate the need to account for the presence/absence of fences in configuration with roads as the results may have repercussions in how we assess the impact of roads on animal movements and distribution. This is important because some species may have behavioral responses and avoid crossing roads, due to the presence of vehicles and the speed they are travelling at. Other species may/may not physically be able to get from one location to the next as they must physically negotiate fences before getting to the road. Therefore, when examining the effects of infrastructure (i.e., roads) on wildlife, researchers and practitioners should account for the presence of other linear features (i.e., fences) within the transportation corridor.

The second difference between movement tactics was resident animals selected areas closer to pasture fences, which contradicted the results for migrant animals. Multiple inferences can be ascertained from this unexpected result. First, resident animals may be occupying areas with higher pasture fence densities and therefore could not distribute themselves away from fences, which resulted in the higher relative selection strength in proximity to pasture fences as seen in Fig. 4. Secondly, resident animals, as a result of remaining on the same range year-round, may have greater site familiarity of their surroundings [70]. It has previously been shown that pronghorn have spatial memory of known fence crossing locations; areas along fences where they know they can cross easily [37]. Resident animals may have greater familiarity to these known crossing sites which allows them to be more comfortable in proximity to fences. Thirdly, fence design may be impeding passage by resident animals creating a semi-permeable barrier effect with resident animals being in proximity to the fence more frequently than migrants, but unable to cross. Therefore, one or several of these fence related inferences may be a factor contributing to the loss of migration by individuals classified as residents. It has been demonstrated that the cost to migrate increases when avoidance of barriers increases [12]. Van Moorter et al. [12] concluded that the proportion of a population undergoing migration would decrease to the extent that the population becomes fully residential when complete avoidance from barriers occurs. While beyond the scope of our study, there is the opportunity to examine the affects of different densities of linear disturbances to determine threshold levels that may result in negative affects to migration and fitness across a population [12, 13].

Many wildlife investigations report the impact of anthropogenic disturbance on wildlife from a specific disturbance type [25]. Few, however, have examined how the spatial configuration between multiple anthropogenic features can influence selection patterns. In one example, crossing rates by caribou (*Rangifer tarandus*) decreased when roads and pipelines were found in tandem and parallel to each other, compared to when they were found separately [71]. In another example, the combination of roads and powerlines resulted in higher barrier effect for moose [2]. Previous studies on pronghorn have shown that resource selection decreases as a function of increased anthropogenic disturbances (e.g., roads, well pads, and fences) [5, 46, 72, 73]. Yet, these studies examined each anthropogenic feature individually and did not include an interactive term in their models. In our analysis, we could have examined each linear feature (i.e., road and fence) as a main effect, as well as including a two-way interaction term (i.e., road x fence) and concluded that roads, fences, and the two-way interaction term effected the selection patterns of pronghorn. However, using an interaction term would not account for the spatial configuration of the boundless matrix of fences and roads across a landscape. For example, an animal crossing two pasture fences and an unfenced road would be analytically equivalent when using an interaction term, to an animal crossing a road fenced on both sides. However, to appropriately capture the underlying crossing behavior of pronghorn requires understanding the spatial configuration of linear features as indicated by our results. The vigilance and energy involved with the physical navigation and behavioral responses associated with crossing a road fenced on both sides is likely more demanding than crossing two pasture fences and a road with no fence. We encourage other researchers to consider behavioral responses by wildlife when deciding to use an interaction term versus constructing spatially explicit covariates that represent the coupling of linear features in their models.

## Conclusion

Our results indicate that in general both fences and roads are impacting pronghorn resource use spatially and behaviorally, whether each linear feature is found separately or in tandem. Modifying existing fences and roads to account for responses to these distinct linear features could facilitate more successful crossing opportunities and/or shifts in space use to utilize mitigated crossing locations [7, 32, 33]. As demonstrated by Jones et al. [5], a 16–38% increase in high quality pronghorn habitat can be achieved by removing (or enhancing) existing fences on the landscape. Enhancements to existing fences to make them wildlife-friendlier would include the replacement of the bottom barbed wire with a double stranded smooth wire at a minimum height of 46 cm [37, 74]. Installing highway overpasses with wing-fencing to funnel individuals is an effective mitigation strategy that allows successful wildlife movement across roads while maintaining vehicle driver safety [33, 75]. To accomplish these mitigations requires collaborating with landowners and transportation agencies to ensure project success. For example, the area in the eastern portion of our study area has been identified as high value for pronghorn connectivity [48]. This area is bisected by the Trans-Canada Highway, with road sections of varying fence configurations (none, one, or both sides) and the surrounding area comprised of differing degrees of pasture fence densities. This is a prime area to install an overpass with wing fencing along strategic road sections and to modify the adjacent pasture fences to facilitate movement [46, 48]. Allowing pronghorn to freely move across the landscape will maintain functional connectivity to ensure population persistence of this endemic ungulate.

## Supplementary Information

**Additional file 1**. Supplementary Information, Tables, and Figures.

## Data Availability

Spatial landcover data are freely available at https://open.canada.ca/data/en/dataset/97126362-5a85-4fe0-9dc2-915464cfdbb7. Pronghorn movement data are also available via Movebank under the name: Northern Sagebrush Steppe Pronghorns. Other datasets used and/or analyzed during the current study are available from the corresponding author upon reasonable request.

## Abbreviations

CI: Confidence interval
FENCE: Pasture fence (fence not associated with a road)
GPS: Global positioning system
SE: Standard error
RNF: Road with no fences
R1F: Road fenced on one side
R2F: Road fenced on both sides
RSS: Relative selection strength
