# Supplementary material for: Is it the road or the fence? Influence of linear anthropogenic features on the movement and distribution of a partially migratory ungulate
Source: Mov Ecol. 2022 Aug 29;10:37. doi: 10.1186/s40462-022-00336-3 (PMC9422137; doi:10.1186/s40462-022-00336-3)
Supplement: Supplementary file 1 — Additional file 1. Supplementary Information, Tables, and Figures. [file 40462_2022_336_MOESM1_ESM.docx]

**Is it the Road or the Fence? Influence of Linear Anthropogenic Features on the Movement and Distribution of a Partially Migratory Ungulate**

**Paul F. Jones, Andrew F. Jakes, Scott E. Vegter, and Mike S. Verhage**

**Supplementary Information, Tables, and Figures**

**Supplementary Information 1.** Steps used to generate the fence, road with no fence, road with 1 fence, and road fenced on both sides spatial data.

Step 1: Separating the Road Layer into 3 Types

- Select by attributes: Paved divided, Paved undivided and gravel, dirt, or truck trail. To separate major highways, secondary highways, and county roads.
- Output created sperate layer for each road type.

Step 2: Create Roadless Fence Layer

- - Ran a 40 m buffer from Major and Secondary highways. The 40 m buffer represents the standard right-of-way area for paved roads in Alberta.
  - Ran 20 m buffer from county roads. The 20 m buffer represents the standard right-of-way area for unpaved roads in Alberta.
  - Intersected fence layer with the buffer layers.
  - Erased the intersecting fence from the original fence layer.
  - Output created the roadless fence layer (pasture fences).

Step 3: Create County Road Layer

- - Note: county road layer also included dirt roads and truck trails.
  - Erased county road intersects, and county road/fence intersects using a 20 m buffer around the intersect point. This made sure that buffers intersecting at corners would not create errors in the data. This created spaces in the fence layer that made errors in the output. County roads in these gaps are displayed as fenceless even when they may have fence on both sides or one.
  - Ran 20 m left and right buffer from the county road.
  - Ran intersect on the buffer and the fence layer creating a left and right fence intersect.
  - Ran 20 m full buffer from the left fence layer and the right fence layer.
  - Then took the county road that intersected with both fence buffers creating the county road with fence on both sides layer.
  - Erased the county two fenced layer from the original county layer.
  - Intersect new county layer with left fence buffer.
  - Intersect new county layer with right fence buffer.
  - Merge those to create the fence with 1 side layer.
  - Erase the county 1 side layer to get the county no fence layer.
  - Output created a layer for county roads not fenced, county roads fenced 1 side, and county road fenced both sides.

Step 4: Create Secondary Highway Layer

- - Used same method as county road.
  - Ran 40 m buffers instead of 20 m.
  - Output created a layer for secondary highway not fenced, secondary highway fenced 1 side, and secondary highway fenced both sides.

Step 5: Create Major Highways Layer

- - Ran 40 m left and right buffer from the major highway.
  - Took parts of fence layer that intersected the buffer.
  - Ran 80 m buffer from left and right fence intersect to ensure it covered both sides of the divided highway.
  - Took major highway that intersected both fence buffers to get major highway with fence on both sides.
  - Erase road with 2 fences.
  - Took major highway that intersected the left and right buffer separately then merged both of those to get major highway with 1 fence.
  - Erased road with 1 fence layer to get major highway with no fences.
  - Output created a layer for major highway not fenced, major highway fenced 1 side, and major highway fenced both sides.

Note:

- County and Secondary layers were cleaned by running the Remove Identical tool and removing line segments < 2 m then running the Snap Tool with a threshold of 2 meters.
- Major Highways were manually cleaned due to their small size.

Step 6: Final Data Creation:

Because of variation in densities between the 10 spatial layers and issues with convergence experienced during exploratory modeling efforts, we were forced to combine covariate data at both scales into 4 linear covariates as follows:

- Fence (hereafter FENCE) data which represents pasture fences.
- Road no fence (hereafter RNF) – combined the data for major highways no fences, secondary highway no fences, and county road no fences.
- Road one fence (hereafter R1F) – combined the data for major highways fenced one side, secondary highway fenced one side, and county road fenced one side.
- Road two fence (hereafter R2F) – combined the data for major highways fenced both sides fence, secondary highway fenced both sides, and county road fenced both sides.
- We combined data for both scales of selection for similar covariates into one covariate. For example, at the fine scale we summed the number of crossings of a major road with 2 fences, a secondary road with 2 fences, and a county road with 2 fences, for each used relocation and available point, to determine the number of crossings of R2F (road with 2 fences). Similarly, at the broad scale we used the minimum distance between a major road with 2 fences, a secondary road with 2 fences, and a county road with 2 fences, for each used relocation and available point, to determine the distance to the R2F (road with 2 fences) covariate. That is if the distances were 100 m, 310 m, and 1000 m for the major road with 2 fences, a secondary road with 2 fences, and a county road with 2 fences respectively, the R2F distance would be 100 m.

**Supplementary Tables:**

**Supplementary Table 1.** Values used to cap the number of crossings for each linear feature for the crossing effect to avoid issues of convergence in the conditional logistic regression model. The 4 linear features were pasture fence (FENCE), a road with no associated fences (RNF), a road fenced on one side (R1F), and a road fenced on both sides (R2F).

| **Covariate** | **Migrant** | **Resident** |
| --- | --- | --- |
| Fence | 10 | 6 |
| RNF | 6 | 4 |
| R1F | 3 | 1 |
| R2F | 2 | 1 |

**Supplementary Table 2.** Top model(s) and next non-competing model for the crossing and proximity effects for migratory and resident pronghorn in Alberta, Canada, 2003–2007. We considered models to be competitive if ΔAIC < 2.0. For the crossing effect models included land cover (Native, Annual crop (reference), Perennial, and Other) plus 4 linear features represented as the number of crossings between consecutive 4 hr relocations. The proximity models included land cover plus the distance too (m) for the 4 linear features. The 4 linear features were pasture fence (FENCE), a road with no associated fences (RNF), a road fenced on one side (R1F), and a road fenced on both sides (R2F).

| **Effect** | **Tactic** | **Model Covariates** | **AIC** | **∆AIC** | **w_i*_** |
| --- | --- | --- | --- | --- | --- |
| Crossing | Migrant | Land cover + FENCE + R2F + R1F + RNF | 68547 | 0.00 | 1.00 |
|  |  | FENCE + R2F + R1F + RNF | 68838 | 291.17 | 0.00 |
|  |  | Land cover | 134153 | 65605.67 | 0.00 |
|  |  | null model | 134859 | 66311.67 | 0.00 |
|  | Resident | Land cover + FENCE + R2F + R1F + RNF | 125381 | 0.00 | 1.00 |
|  |  | FENCE + R2F + R1F + RNF | 126773 | 1392.30 | 0.00 |
|  |  | Land cover | 199823 | 74441.60 | 0.00 |
|  |  | null model | 202827 | 77446.30 | 0.00 |
| Proximity | Migrant | Land cover +FENCE + R2F + R1F + RNF | 135149 | 0.00 | 0.78 |
|  |  | Land cover + FENCE + R1F + RNF | 135151 | 2.50 | 0.22 |
|  |  | Land cover | 135428 | 278.80 | 0.00 |
|  |  | null model | 135701 | 551.80 | 0.00 |
|  | Resident | Land cover + FENCE + RNF | 201751 | 0.00 | 0.31 |
|  |  | Land cover + FENCE + RNF + R1F | 201752 | 0.80 | 0.21 |
|  |  | Land cover + RNF + R1F | 201753 | 1.60 | 0.14 |
|  |  | Land cover + FENCE + RNF + R2F | 201753 | 1.90 | 0.12 |
|  |  | Land cover + FENCE + RNF + R1F + R2F | 201754 | 2.80 | 0.08 |
|  |  | Land cover | 202860 | 1108.90 | 0.00 |
|  |  | null model | 203963 | 2211.70 | 0.00 |

* model weight

**Supplementary Figures:**

**
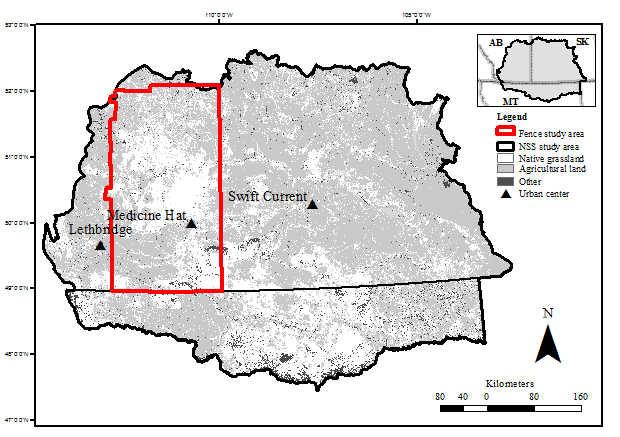
**

**Supplementary Fig. 1.** Linear feature study area within the Alberta portion of the larger Northern Sagebrush Steppe study area that encompasses Alberta and Saskatchewan, Canada and Montana, USA, 2003-2007. The solid red line boundary represents the area where fence spatial data was available.


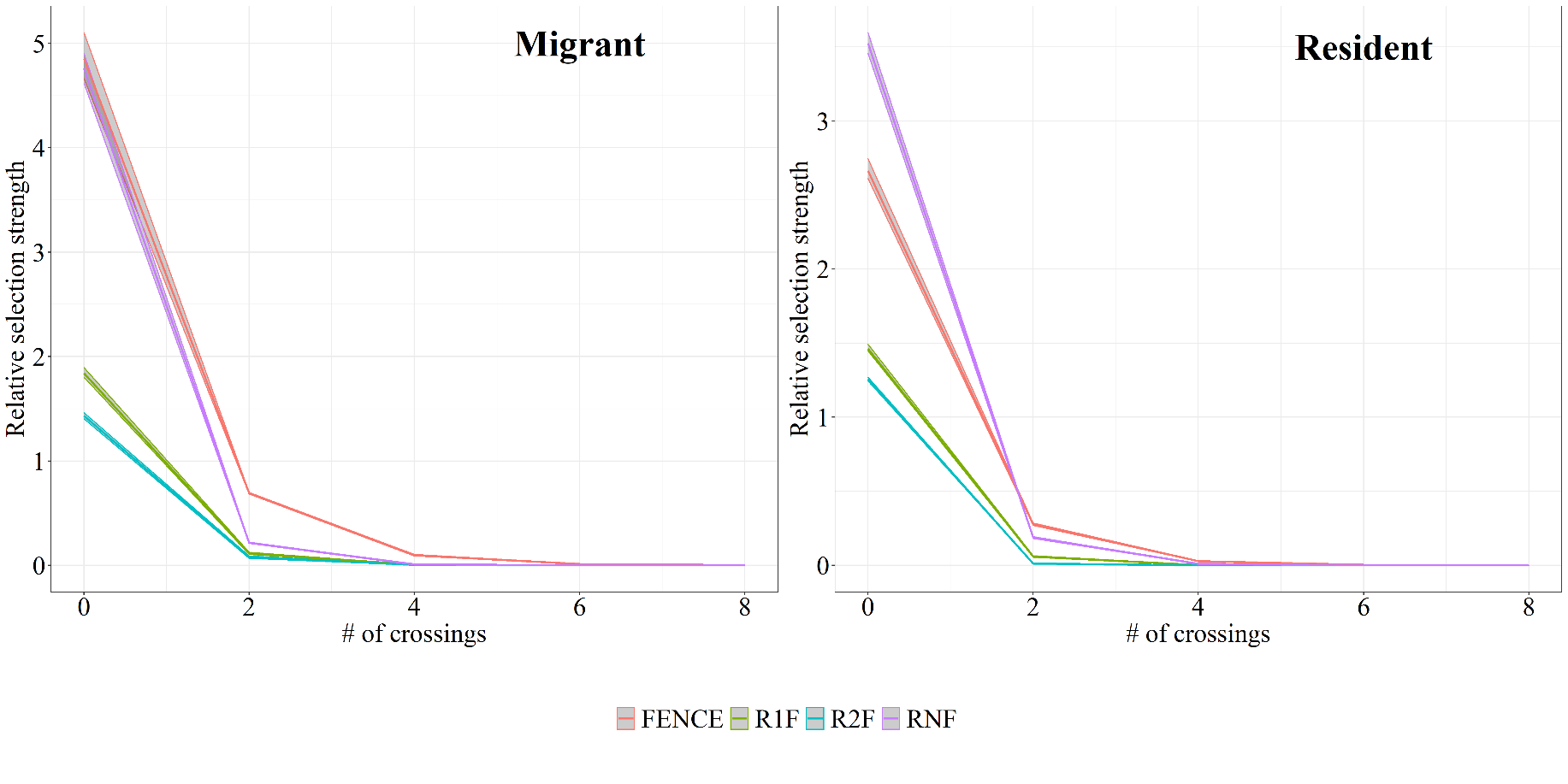


**Supplementary Fig. 2.** Relative selection strength (exponential of the log-RSS) for the covariates in the top conditional logistic model for crossing effect for migrant (left) and resident pronghorn (right) in Alberta, Canada, 2003–2007. The RSS was calculated by comparing the number of crossings across the range of capped values for each covariate to the mean number of crossings of that covariate, while holding the other covariates in the model constant at their mean and setting land cover to Native. The 4 linear covariates are pasture fence (FENCE), a road with no associated fences (RNF), a road fenced on one side (R1F), and a road fenced on both sides (R2F).
